# Cutaneous Rosai-Dorfman-Destombes disease: the diagnostic value of the yellow island sign

**DOI:** 10.1093/omcr/omag102

**Published:** 2026-06-21

**Authors:** Maha El Maati, Fatima Zahra Sassine, Mouna Rimani, Leila Benzekri, Nadia Ismaili

**Affiliations:** Dermatology Department, Ibn Sina University Hospital, Mohammed V University, United Nations Avenue, Rabat 10000, Morocco; Dermatology Department, Ibn Sina University Hospital, Mohammed V University, United Nations Avenue, Rabat 10000, Morocco; Pathology Department, 6 Derna street, Center of Hassan, Rabat 10020, Morocco; Dermatology Department, Ibn Sina University Hospital, Mohammed V University, United Nations Avenue, Rabat 10000, Morocco; Dermatology Department, Ibn Sina University Hospital, Mohammed V University, United Nations Avenue, Rabat 10000, Morocco

**Keywords:** cutaneous Rosai-Dorfman-Destombes disease, Dermoscopy, emperipolesis, Histiocytosis, surgical excision

## Abstract

Rosai-Dorfman-Destombes (RDD) disease is a rare non-Langerhans cell histiocytosis typically characterized by massive lymphadenopathy. The purely cutaneous subtype (CRDD) is clinically polymorphic, mimicking various inflammatory or neoplastic conditions, making its diagnosis challenging. We present a case of a young phototype 4 Moroccan female with a 2-year history of a progressively enlarging violaceous tumor on her posterior thigh. Dermoscopy revealed structureless yellow areas with fine telangiectasias. Histological along with immunohistochemical examination showed a dense dermal infiltrate with large histiocytes exhibiting prominent emperipolesis confirming the diagnosis of CRDD. A comprehensive systemic workup was negative. No recurrence was detected after a 6 month follow up after surgical excision. This case highlights the importance of dermoscopic clues which correlates with the histological findings. In patients with higher phototypes, these findings are essential to guide the clinician toward a histiocytic disorder. Differential diagnoses such as Langerhans cell histiocytosis, juvenile xanthogranuloma, and sarcoidosis must be ruled out through immunohistopathology.

## Introduction

Rosai-Dorfman-Destombes (RDD) disease is a rare non-Langerhans cell histiocytosis, typically characterized by massive, painless cervical lymphadenopathy occurring mostly in young African male [1]. While the systemic form is well-documented, the purely cutaneous subtype (CRDD) is an exceptional entity that clinicians must not overlook [2]. CRDD often presents without the systemic symptoms or lymph node involvement characteristic of the classic form [3]. Given its significant clinical polymorphism, it can mimic various inflammatory or neoplastic conditions, making its diagnosis a challenge. We report a case of a 25-year-old Moroccan female presenting with a localized, polylobed tumour on the thigh.

## Case presentation

**Figure 1:**
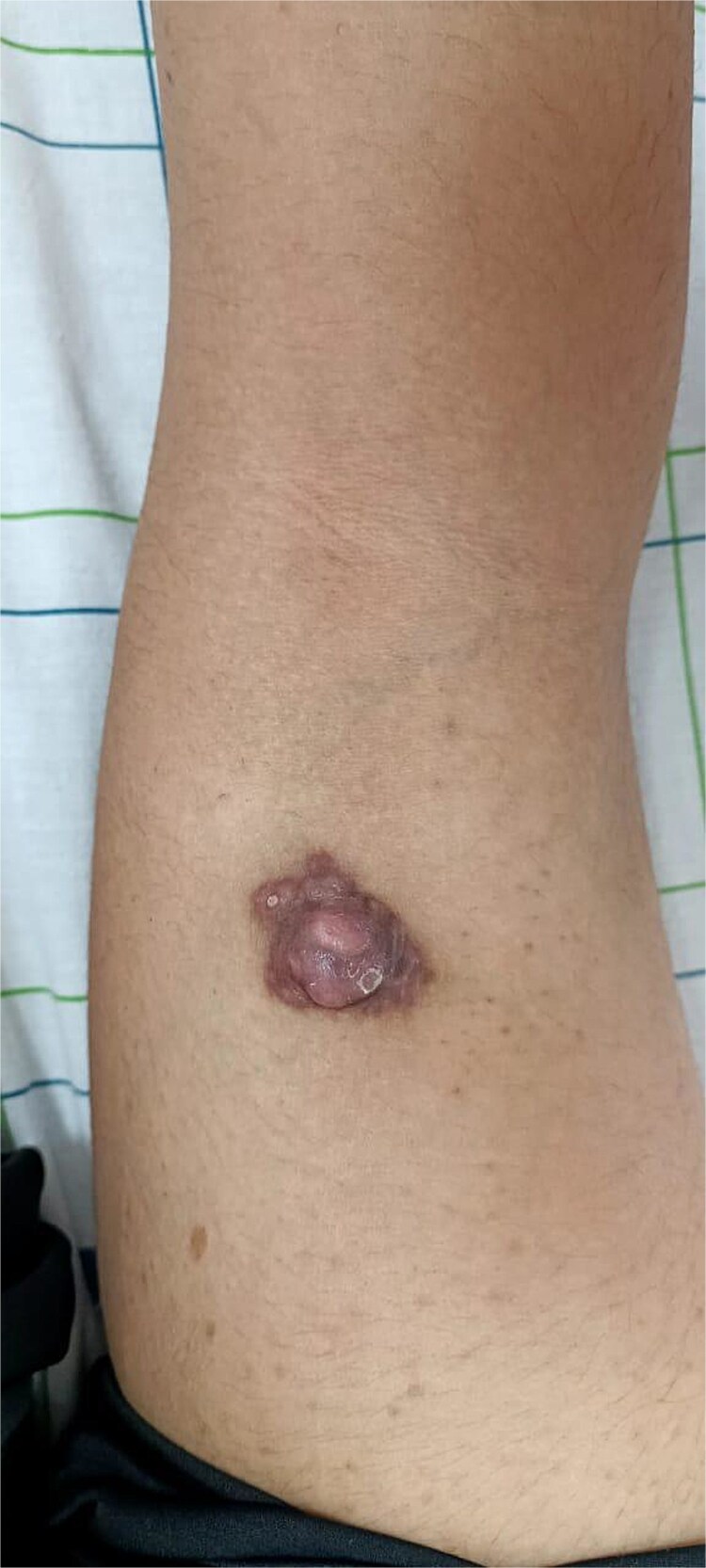
Polylobed, brownish-violaceous tumour with focal yellowish areas on the posterior thigh.

**Figure 2:**
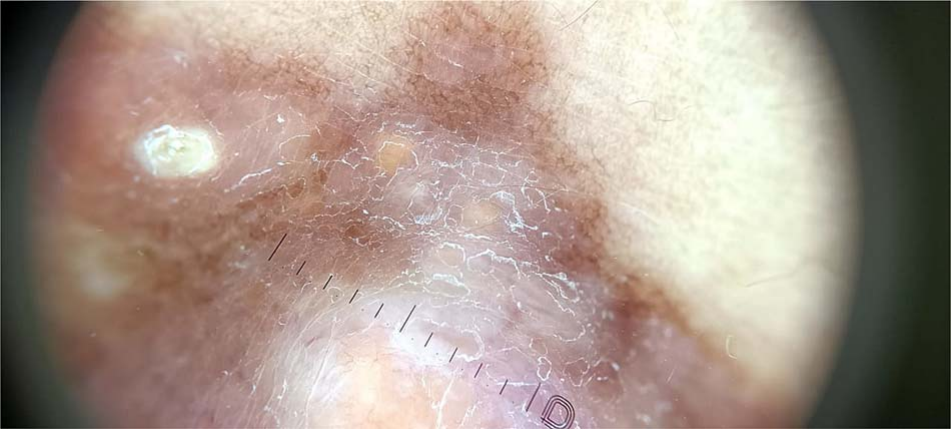
Dermoscopy showing structureless yellow areas on a brownish-violaceous background, fine telangiectasias, and a peripheral pigment network.

**Figure 3:**
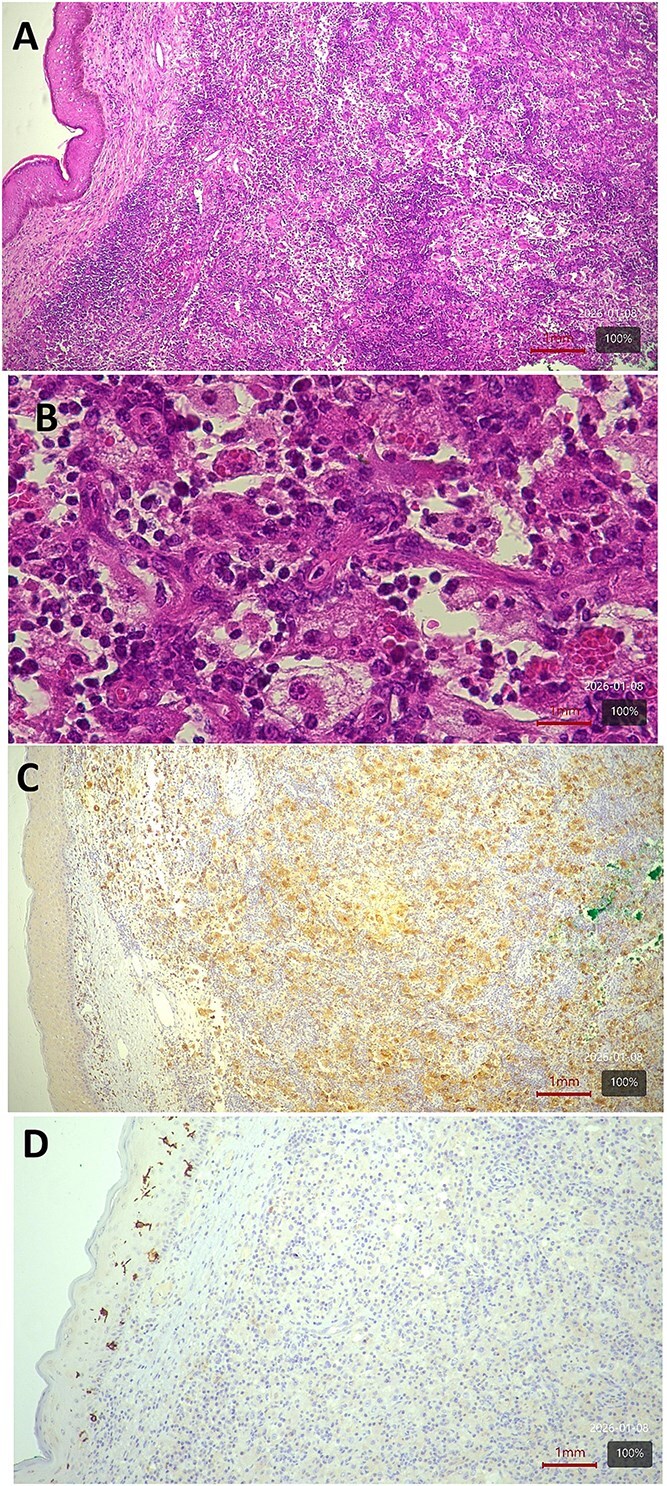
(A) Dense and diffuse infiltration of the reticular dermis with clear areas corresponding to clusters of histiocytes mixed with lymphocytes and plasma cells. (B) Emperipolesis phenomenon. (C) CD163 (+) histiocytes. (D) CD1a (−) histiocytes, control of intraepidermal Langerhans cells (+).

A 25-year-old Moroccan female (Fitzpatrick skin phototype IV) with an unremarkable medical history presented with a 2-year history of a progressively enlarging tumour on her posterior thigh. Clinical examination revealed a 2.5 cm soft, polylobed, violaceous tumour with yellowish areas. The surface was smooth with focal scaling (Fig. 1). Dermoscopy showed structureless yellow areas on a brownish-violaceous background, fine telangiectasias, and a peripheral pigment network (Fig. 2). Histological analysis of the lesion revealed a dense pseudo-tumoral dermal infiltrate composed of small lymphocytes, mature plasma cells, and neutrophils. Within the infiltrate, large vacuolated histiocytes exhibited significant emperipolesis. PAS and Gram stains were negative. Immunohistochemistry confirmed the diagnosis, with histiocytes staining positive for S100 and CD163, and negative for CD1a (Fig. 3). A systemic workup, including a CT scan and long-bone X-rays, showed no abnormalities. The patient underwent complete surgical excision of the remaining lesion with a favourable outcome.

## Discussion

Purely CRDD disease is a distinct clinical entity. Characterized by an average onset at 20.6 years, the condition primarily involves children and young adults. Clinical data also reflect a higher incidence in African patients with a discrete male preponderance (sex ratio 1.4) [1–3]. Dermoscopic findings in this case are highly characteristic. The ‘structureless yellow areas’ correlate histologically with the underlying dense histiocytic infiltration and lipid deposits within the dermis [4, 5]. These yellow zones, combined with fine telangiectasias, are valuable clues for orienting the diagnosis toward a granulomatous or histiocytic process, especially in patients with higher phototypes where the background may appear more violaceous [5]. The clinical diagnosis of CRDD disease is challenging due to its ability to mimic various granulomatous or neoplastic processes [2]. In our case, the identification of “structureless yellow areas” (the “yellow island” sign) was a pivotal dermoscopic clue. These areas correlate histologically with the massive dermal infiltration of histiocytes and the presence of lipid deposits [4, 5]. While this sign is frequently described in granulomatous diseases, its association with fine, branching telangiectasias in a tumoral lesion should strongly orient the clinician toward a histiocytic disorder [5]. The definitive diagnosis of CRDD remains strictly immunohistopathological. The hallmark feature is emperipolesis [6]. In our patient, this feature was prominently observed within a background of mature plasma cells and lymphocytes, which is consistent with the international consensus diagnostic criteria [1]. The differential diagnosis must be done with the other Langerhans Cell Histiocytosis that can present with cutaneous nodules, it is characterized histologically by “coffee-bean” shaped nuclei and, crucially, an immunohistochemical profile that is positive for CD1a and Langerin. In our case, the CD1a negativity was essential to exclude LCH [1, 6].

Juvenile xanthogranuloma often presents with similar yellowish clinical and dermoscopic features. However, histologically, JXG histiocytes (including Touton giant cells) are typically S100 negative [6]. Cutaneous Sarcoidosis must also be ruled out, it can display yellow-orange areas on dermoscopy, it is characterized by “naked” non-caseating granulomas without the prominent emperipolesis or the specific plasma-cell-rich infiltrate seen in CRDD [4, 5]. Infectious Granuloma must also be ruled out given the patient’s origin and the clinical appearance [1].

Consistent with international consensus recommendations, a thorough systemic workup is mandatory to ensure the disease is limited to the skin [1] the absence of lymphadenopathy and the negative systemic workup (CT scan and bone X-rays) confirmed that this was a purely cutaneous localized form, which generally carries a much more favourable prognosis than the systemic or multi-organ variants [2, 3].

Management of CRDD is not standardized due to its rarity. Options range from observation (as some cases undergo spontaneous regression) to corticosteroids, radiotherapy, or surgery. In this case, complete surgical excision resulted in a favourable outcome with no recurrence, supporting the efficacy of surgery for localized tumours. For localized CRDD, surgical excision remains an excellent therapeutic option, often resulting in complete resolution [4].

## IRB approval status

Not applicable.
